# Structural and Biophysical Insights into the Role of CD4 and CD8 in T Cell Activation

**DOI:** 10.3389/fimmu.2013.00206

**Published:** 2013-07-22

**Authors:** Yili Li, Yiyuan Yin, Roy A. Mariuzza

**Affiliations:** ^1^W. M. Keck Laboratory for Structural Biology, Institute for Bioscience and Biotechnology Research, University of Maryland, Rockville, MD, USA; ^2^Department of Cell Biology and Molecular Genetics, University of Maryland, College Park, MD, USA; ^3^Department of Antibody Engineering, Genentech, South San Francisco, CA, USA

**Keywords:** CD4, CD8, MHC, T cell receptor, structure, T cell activation

## Abstract

T cell receptors (TCRs) recognize peptides presented by MHC molecules (pMHC) on an antigen-presenting cell (APC) to discriminate foreign from self-antigens and initiate adaptive immune responses. In addition, T cell activation generally requires binding of this same pMHC to a CD4 or CD8 co-receptor, resulting in assembly of a TCR–pMHC–CD4 or TCR–pMHC–CD8 complex and recruitment of Lck via its association with the co-receptor. Here we review structural and biophysical studies of CD4 and CD8 interactions with MHC molecules and TCR–pMHC complexes. Crystal structures have been determined of CD8αα and CD8αβ in complex with MHC class I, of CD4 bound to MHC class II, and of a complete TCR–pMHC–CD4 ternary complex. Additionally, the binding of these co-receptors to pMHC and TCR–pMHC ligands has been investigated both in solution and *in situ* at the T cell–APC interface. Together, these studies have provided key insights into the role of CD4 and CD8 in T cell activation, and into how these co-receptors focus TCR on MHC to guide TCR docking on pMHC during thymic T cell selection.

## Introduction

Adaptive immunity depends on specific recognition by an αβ T cell receptor (TCR) of an antigenic peptide bound to a major histocompatibility complex (pMHC) molecule on an antigen-presenting cell (APC). However, TCR–pMHC interactions alone do not efficiently trigger T cells, requiring the participation of the co-receptors CD4 and CD8 (1–4). These transmembrane glycoproteins mark different T cell subsets. CD4 is expressed on Th1, Th2, and Th17 helper cells, as well as on CD4 regulatory T cells (Tregs); CD8 is expressed on cytotoxic T lymphocytes (CTLs) and CD8 Tregs. CD4 and CD8 enhance T cell signaling by binding MHC class II (CD4) or MHC class I (CD8) molecules on APCs. The interaction of CD4 with MHC class II greatly reduces the number of antigenic peptides required for T cell activation (5) and substantially increases cytokine production by helper T cells (1). Likewise, the CD8–MHC class I interaction enhances the antigen sensitivity and response of cytotoxic T cells to pMHC ligands (6).

The principal role of the CD4 and CD8 co-receptors is to recruit the Src tyrosine kinase p65lck (Lck) to the TCR–pMHC complex following co-receptor binding to MHC, resulting in assembly of a TCR–pMHC–CD4 or TCR–pMHC–CD8 ternary complex (7–10). Recruitment of Lck occurs via its association with the cytoplasmic tail of CD4 or CD8. The accompanying increase in the local concentration of Lck promotes phosphorylation of immunoreceptor tyrosine activation motifs (ITAMs) in the cytoplasmic tails of CD3 subunits associated with the TCR in the TCR–CD3 complex, leading to the recruitment and activation of Zap-70. Activated Zap-70 phosphorylates LAT and SLP-76, which function as scaffolds to recruit other signaling molecules to the downstream T cell signaling apparatus that regulates T cell activation, proliferation, and differentiation. The targeted delivery of Lck to TCR by CD4 or CD8 during thymic selection is believed to impose MHC restriction on the developing αβ TCR repertoire (11). In support of this idea, αβ TCRs in mice lacking co-receptors and MHC are not biased toward pMHC ligands, but instead display antibody-like recognition specificities (12, 13).

This review focuses on structural and biophysical studies of CD4 and CD8 interactions with MHC molecules. Crystal structures have been determined of CD8αα and CD8αβ in complex with MHC class I, of CD4 bound to MHC class II, and, most recently, of a complete TCR–pMHC–CD4 ternary complex. In addition, the binding of these co-receptors to pMHC and TCR–pMHC ligands has been investigated both in solution and *in situ*. Collectively, these studies have provided critical insights into the role of CD4 and CD8 in T cell activation and in guiding TCR docking on pMHC during T cell development.

## Interaction of CD8 with MHC Class I

CD8 exists as two isoforms, CD8αα and CD8αβ, which are expressed on different cell types and appear to have different functions. CD8ββ homodimers have also been reported, but these lack MHC-binding activity and their significance is unknown (14). Whereas CD8αα is found on γδ T cells, intestinal intraepithelial T lymphocytes, and natural killer (NK) cells, CD8αβ is expressed on αβ TCR thymocytes and CTLs. The function of CD8αα remains obscure, but it has been implicated in the negative regulation of intestinal intraepithelial T lymphocytes (15). By contrast, CD8αβ is clearly required for positive selection of CD8^+^ T cells in the thymus (16–18) and for activation of CD8^+^ T cells in the periphery (19). In both CD8αα and CD8αβ, Lck associates with the CD8α chain. In addition, ligation of CD8αβ by MHC, in the absence of TCR engagement, results in apoptosis of a proportion of double-positive thymocytes, which may be a mechanism for removing thymocytes that have failed positive selection (20).

Fluorescence resonance energy transfer experiments have shown that, during T cell activation, the TCR binds initially to pMHC, and that CD8 then binds to the same pMHC as the TCR, leading to formation of a TCR–pMHC–CD8 complex (21). This order of engagement ensures that the specific TCR–pMHC interaction dominates T cell recognition. As measured by surface plasmon resonance (SPR), CD8 binds very weakly to MHC class I in both mouse and human, with little influence of the MHC-bound peptide (21–23). In the mouse, the average binding affinity for CD8αβ (*K*_D_ ∼50 μM) is similar to that for CD8αα (∼70 μM), with some variation depending on the particular MHC class I allele. The human CD8–MHC class I interaction is weaker still, averaging ∼150 μM (24–26). In both cases, these affinities are 10- to 100-fold lower than for most TCR–pMHC interactions.

In overall agreement with these SPR results, *in situ* measurements of the mouse CD8–MHC class I interaction gave an affinity several orders of magnitude lower than that of TCR for pMHC (27). These experiments used a micropipette adhesion frequency assay to measure the adhesion kinetics of live T cells interacting with pMHC ligands presented on surrogate APCs. The two-dimensional (2D) binding parameters derived from this technique are thought to more accurately reflect biological interactions in membranes than the three-dimensional (3D) parameters derived from SPR, in which fluid-phase receptors and ligands are removed from their cellular environment (28).

Although the affinity of CD8 for MHC class I is weak, recent 2D affinity measurements support the idea that CD8 contributes significantly to stabilizing the TCR–pMHC interaction at the T cell–APC interface (9). These experiments revealed that the TCR–pMHC–CD8 trimolecular interaction generates synergy over the simple sum of the individual TCR–pMHC and pMHC–CD8 interactions, and that this cooperativity amplifies peptide discrimination. Thus, in addition to its primary role of recruiting Lck to the TCR–pMHC complex, a secondary function of CD8 is to reinforce TCR binding to the pMHC ligand. Whether the CD4 co-receptor also promotes cooperative binding remains to be determined.

The micropipette adhesion frequency assay also revealed that the kinetics of the TCR–pMHC–CD8 trimolecular interaction at the T cell membrane proceeds in two stages (9). The first consists of TCR-dominant binding to agonist pMHC. This triggers a second stage involving an upregulation of CD8-dependent adhesion after a 1 s delay. The second stage requires Lck kinase activity to initiate CD8 binding to the same pMHC ligand engaged by the TCR, generating synergy. It remains to be established whether the TCR–pMHC–CD4 trimolecular interaction involves a similar sequence of events.

## Structures of CD8 Bound to MHC Class I

CD8αβ is a heterodimeric type I transmembrane glycoprotein, whose α and β chains are each composed of an immunoglobulin (Ig)-like domain connected by a long stalk to a transmembrane domain and a cytoplasmic tail, with Lck bound to the CD8α tail. By contrast, the CD8αα homodimer comprises only the α chain. Four structures of CD8αα or CD8αβ bound to MHC class I molecules have been reported: (1) the complex between human CDαα and HLA-A^∗^0201 (29); (2) the complex between human CD8αα and HLA-A^∗^2402 (30); (3) the complex between mouse CD8αα and H-2K^b^ (31); and (4) the complex between mouse CD8αβ and H-2D^d^ (23).

**Figure 1:**
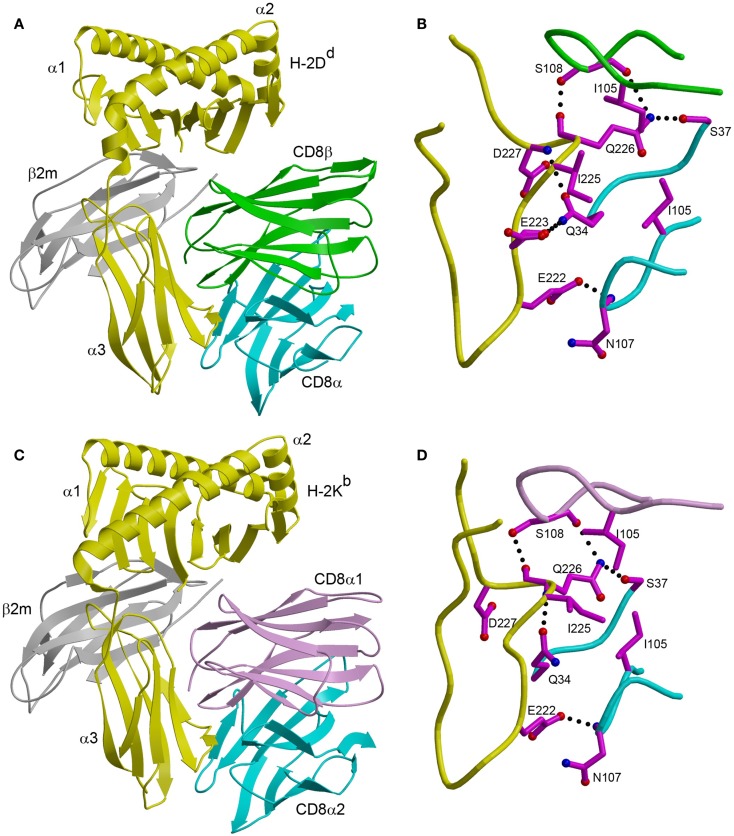
**Comparison of CD8αβ–H-2D^d^ and CD8αα–H-2K^b^ complexes**. **(A)** Ribbon diagram of the CD8αβ–H-2D^d^ complex (Protein Data Bank accession code 3DMM) (23). MHC α chain, yellow; β_2_m, gray; CD8α, cyan; CD8β, green. **(B)** Interaction between the CDR-like loops of CD8αβ and the H-2D^d^ α3 CD loop. The side chains of contacting residues are shown in ball-and-stick representation with carbon atoms in magenta, nitrogen atoms in blue, and oxygen atoms in red. Hydrogen bonds are drawn as dotted black lines. **(C)** Ribbon diagram of the CD8αα–H-2K^b^ complex (1BQH) (31). MHC α chain, yellow; β_2_m, gray; CD8α1, pink; CD8α2, cyan. **(D)** Interaction between the CDR-like loops of CD8αα and the H-2K^b^ α3 CD loop.

In the CD8αβ–H-2D^d^ complex (23), the CD8αβ heterodimer contacts only the α3 domain of the MHC class I heavy chain (Figure 1A). By contrast, CD8αα also contacts the α2 domain and β_2_-microglobulin (β_2_m) in the CDαα–HLA-A^∗^0201 (29), CD8αα–HLA-A^∗^2402 (30), and CD8αα–H-2K^b^ complexes (31) (Figure 1C). The CD8β subunit occupies a position equivalent to that of the CD8α1 subunit in the three CD8αα–MHC class I structures, which places CD8β proximal to the T cell membrane. The CD8α subunit of CD8αβ is located in the same position as the CD8α2 subunit, distal from the T cell and near the C-terminus of the MHC class I α3 domain (Figures 1A,C). Nearly all MHC class I residues that mediate key interactions with CD8αα or CD8αβ are non-polymorphic, which explains the largely allele-independent nature of CD8 binding.

For both CD8αα and CD8αβ, the main binding interaction is with a protruding loop in the α3 domain of the MHC class I molecule (CD loop), corresponding to residues 220–228 (Figures 1B,D). This loop is flexible in the absence of CD8, but is stabilized by CD8 binding. In the CD8αα–MHC class I structures, the two CDR3-like loops of the CD8α subunits clamp onto the central β-turn portion of the CD loop in an antibody-like manner, as do the two CDR3-like loops of CD8αβ (23, 29–31). In addition, CD8αα contacts the α2 domain and β_2_m through its CD8α1 subunit, whereas the corresponding CD8β subunit of CD8αβ contacts only α3. Of particular note in the CD8αβ–H-2D^d^ complex is Gln226 of H-2D^d^, a highly conserved residue among MHC class I alleles and the only one that interacts with both CD8α and CD8β (Figure 1B).

## Role of the CD8 Stalk Region in Co-Receptor Function

The Ig-like domains of CD8α and CD8β are tethered to the T cell membrane by long stalk regions comprising ∼45 residues for CD8α and ∼35 residues CD8β (32). These stalk regions, which are rich in threonine, serine, and proline residues, are heavily *O*-glycosylated at multiple sites in all species studied (33). The stalk of CD8αβ undergoes developmentally programed *O*-glycan modification controlled by the sialyltransferase ST3 Gal-I, which catalyzes addition of sialic acid to core 1 *O*-linked glycans (34). In particular, immature CD4^+^CD8^+^ thymocytes exhibit lower levels of CD8 sialylation than mature thymocytes (32, 34, 35). Decreased sialylation of the CD8αβ stalk was found to markedly increase the affinity of CD8 for MHC class I, as measured by MHC tetramer binding, thereby affecting T cell selection (34, 35). Enhanced CD8αβ binding at the CD4^+^CD8^+^ stage facilitates elimination of autoreactive T cells in the thymus. However, once a double-positive thymocyte has differentiated to the CD8^+^ stage, *O*-glycan sialylation of the CD8αβ stalk reduces co-receptor affinity for pMHC, requiring a stronger TCR–pMHC interaction for T cell activation in the periphery (34, 35).

The structural basis for reduced CD8αβ affinity for MHC class I upon sialylation of the stalk region is unknown. One possibility is that sialylation alters the association or orientation of the Ig-like domains of the CD8αβ heterodimer, reducing its capacity to bind MHC class I (34). However, Merry et al. (36) found that sialylation had little or no effect on the overall structure of CD8, insofar as sialylated and non-sialylated forms of soluble CD8αα exhibited indistinguishable hydrodynamic properties. This suggests that the results of Moody et al. (34) and Daniels et al. (35) might be explained by avidity effects arising from aggregation of unsialylated CD8 on the T cell surface that increase MHC tetramer binding (36).

Irrespective of the underlying mechanism, the finding that developmentally regulated glycosylation of the CD8αβ stalk can modulate MHC binding at the cell surface demonstrates that the stalk has a specialized role in co-receptor function, beyond simply attaching the MHC-binding domains to the T cell membrane. The long CD8αβ stalk may also allow a possible *cis* interaction between CD8 and MHC class I expressed on the same T cell (37), in addition to the established *trans* interaction between CD8 and MHC class I expressed on different cells. Interestingly, the stalk regions of other immune system receptors have also been recently found to play prominent roles in receptor function. For example, the long stalk regions of Ly49 NK cell receptors enable binding to MHC class I in *cis* or *trans* configurations (38). Additionally, the stalk domain of the activating NK receptor NKp30 is critical for NK cell killing and may contribute directly to binding its tumor cell ligand B7-H6 (39).

No 3D structural information is available for the stalk regions of CD8αβ or CD8αα, since the stalks, when included in the constructs used for protein expression, were mostly or entirely disordered in the CD8–MHC class I crystal structures (23, 29–31). Although this has been interpreted to mean that that the CD8 stalk is highly flexible, it should be emphasized that the stalks completely lacked glycosylation because the CD8 proteins were produced in bacteria. However, there is biophysical evidence that *O*-glycosylation may significantly restrict the flexibility of the stalks. Studies of mucins have demonstrated that *O*-glycans stiffen polypeptides through steric interactions between peptide-linked *N*-acetylgalactosamine residues and adjacent peptide residues (40, 41). Moreover, *O*-glycans in the CD8 stalk polypeptides were found to reduce the overall extension of the stalk from a theoretical maximum of 3.4 Å per residue to 2.6 Å per residue, indicating rigidification (36, 42). Therefore, *O*-glycosylation may limit the mobility of the CD8 head group relative to the T cell membrane, an important consideration in evaluating co-receptor interactions with TCR–pMHC during thymic selection and peripheral antigen recognition, as discussed below.

## Structures of CD4 Bound to MHC Class II

CD4 is a monomeric type I transmembrane glycoprotein consisting of four Ig-like extracellular domains connected by a short stalk to a transmembrane domain and a cytoplasmic tail that interacts with Lck. CD4 binds MHC class II with exceptionally low affinity compared to all other leukocyte cell–cell recognition molecules characterized to date, whose *K*_D_s typically fall in the 1–100 μM range, as measured by SPR (43, 44). For the CD4–MHC class II interaction, *K*_D_s have been estimated to range from ∼200 μM (for human CD4 binding to mouse MHC class II) (45) to>2 mM (for human CD4 binding to human MHC class II) (43). These affinities are substantially weaker than those for CD8–MHC class I interactions, which range from ∼10 μM (for mouse CD8αβ binding to H-2D^d^) (23) to ∼150 μM (for human CD8αα binding to HLA-A^∗^0201) (26). Presumably, evolution has calibrated the affinity of CD4 for MHC class II to enable peripheral T cells to respond efficiently to the very low abundance of foreign pMHC molecules on APCs (sensitivity), yet avoid activation by the far greater number of self-pMHC molecules (discrimination), which could cause autoimmunity.

The ability of CD4 to recognize highly polymorphic MHC class II molecules is central to its function as a co-receptor (1). In humans, MHC class II molecules are encoded by three separate loci (HLA-DR, -DQ, and -DP), while in mice they are encoded by two loci (I-A and I-E). For HLA-DR, most variability derives from the β-chain, with>700 known alleles at the population level, whereas there are only three α-chain variants. In contrast, both α- and β-chains of HLA-DQ and -DP are polymorphic (46). Two structures of CD4 bound to MHC class II molecules have been reported: (1) the complex between human CD4 (domains D1 and D2) and mouse I-A^k^ to 4.3 Å resolution (47); and (2) the complex between human CD4 (domains D1 and D2) and human HLA-DR1 to 2.4 Å resolution (48). These structures readily explain the ability of CD4 to recognize multiple MHC class II alleles.

**Figure 2:**
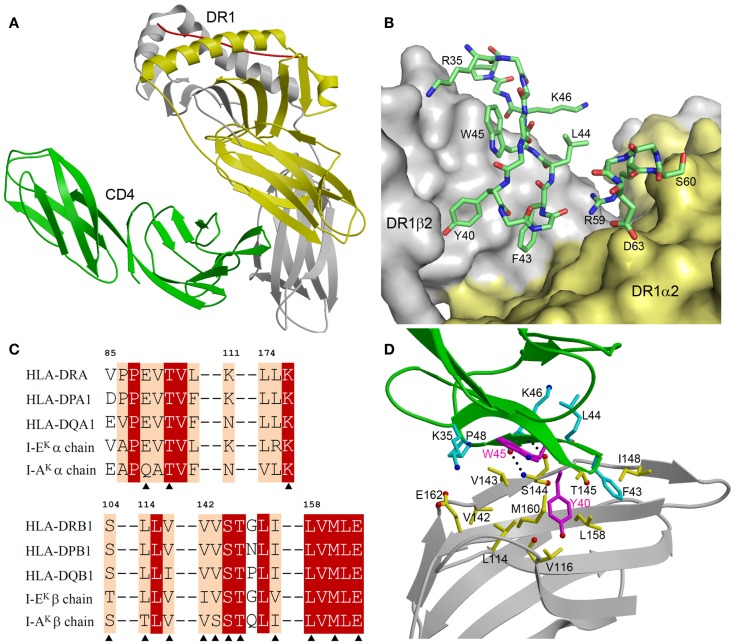
**Structure of a human CD4–MHC class II complex**. **(A)** CD4 (green) contacts the α2 (yellow) and β2 (gray) domains of HLA-DR1 through its D4 domain (3S4S) (48). The MHC-bound peptide is red. **(B)** The CD4–HLA-DR1 binding interface. The two regions of CD4 (residues 35–48 and 59–63) that contact HLA-DR1 are shown in stick representation with carbon atoms in green, oxygen atoms in red, and nitrogen atoms in blue. The molecular surface of HLA-DR1 that interacts with CD4 is depicted with the α2 domain in yellow and the β2 domain in gray. **(C)** Sequence alignment of the CD4-contacting regions of the α- and β-chains of different human and mouse MHC class II alleles. Residues that contact CD4 in the CD4–HLA-DR1 structure are marked with triangles. White characters on a red background show residues that are strictly conserved across human or mouse MHC class II molecules. Black characters on a tan background are conservatively substituted residues. **(D)** Close-up view of the interactions between an affinity-matured CD4 mutant (green) and the HLA-DR1 β2 domain (gray). The side chains of interacting residues are shown in ball-and-stick representation with carbon atoms in cyan (CD4) or yellow (HLA-DR1), oxygen atoms in red, and nitrogen atoms in blue. The mutated Tyr40 and Trp45 residues of CD4 are in magenta. Hydrogen bonds are drawn as dotted black lines.

The CD4–I-A^k^ and CD4–HLA-DR1 complexes display similar overall topologies, although only the higher-resolution CD4–HLA-DR1 structure permitted an atomic-level description of the CD4–MHC class II interface. In both complexes, CD4 binds MHC class II through its membrane-distal D1 domain, which contacts the membrane-proximal α2 and β2 domains of the MHC class II molecule (Figure 2A). CD4 uses two discontinuous regions to engage MHC class II in a concavity formed by the α2 and β2 domains (Figure 2B). The first region, composed of β-strands C′ and C″, exclusively contacts the β2 domain. The second region, comprising a short 3_10_ helix within the loop connecting β-strands D and E, binds solely to the α2 domain.

Figure 2C shows sequence alignments of the α- and β-chains of selected HLA-DR, -DP, and -DQ alleles in the regions where HLA-DR1 contacts CD4. For the β-chains, 11 of 12 CD4-contacting residues are absolutely conserved in these human MHC class II molecules, and 1 is conservatively substituted (Val116Ile in HLA-DQ). For the α-chains, all three CD4-contacting residues (Glu88, Thr90, Leu176) are invariant across human MHC class II alleles. Hence, the remarkable cross-reactivity of CD4 is attributable to the targeting of non-polymorphic residues in the concavity formed by the α2 and β2 domains of HLA-DR, -DP, and -DQ. For I-A^k^, 11 of 14 CD4-contacting residues are identical to those of HLA-DR1, while for I-E^k^ 12 of 14 are identical (Figure 2C). All non-identical residues are conservatively substituted in both molecules. Therefore, CD4 almost certainly engages all human and mouse MHC class II molecules in the same manner as it does HLA-DR (48).

## Structure of a TCR–pMHC–CD4 Ternary Complex

The low affinity of CD4 for MHC class II presented a major technical obstacle to crystallizing a TCR–pMHC–CD4 ternary complex. To overcome this obstacle, *in vitro* directed evolution by yeast surface display was used to increase the affinity of CD4 for MHC class II (49). Affinity maturation by yeast display relies on expression of a library of mutants on the surface of yeast, followed by selection of variants with improved affinity (50). The D1 domain of human CD4 was subjected to *in vitro* random mutagenesis, and the resulting mutant library was displayed on yeast by fusion to agglutinin protein Aga2p (48). Because CD4 binds MHC class II very weakly (43, 45), the CD4 library was sorted by flow cytometry using HLA-DR1 tetramers, rather than monomers, to increase the avidity of the selecting ligand. In this way, a CD4 mutant that bound HLA-DR1 with *K*_D_ = 9 μM was isolated, compared with no detectable binding for wild-type CD4, even at high concentrations (400 μM). The CD4 mutant exhibited similar affinity for HLA-DR4 (*K*_D_ = 10 μM), in agreement with the ability of CD4 to recognize all HLA-DR alleles, as discussed above.

The affinity-matured CD4 mutant contained two substitutions in the D1 domain: Gln40Tyr and Thr45Trp. In the mutant CD4–HLA-DR1 structure (48), CD4 Trp45 is located at the center of the interface with HLA-DR1, where its bulky side chain makes multiple hydrophobic contacts with DR1 β2 Val143 (Figure 2D). Similarly, CD4 Tyr40 is surrounded by apolar DR1 β2 residues Leu114, Val116, Leu158, and Met160, resulting in increased hydrophobic interactions at the mutation site. Together, the Gln40Tyr and Thr45Trp mutations improved shape and chemical complementarity with HLA-DR1, thereby stabilizing the CD4–HLA-DR1 complex.

**Figure 3:**
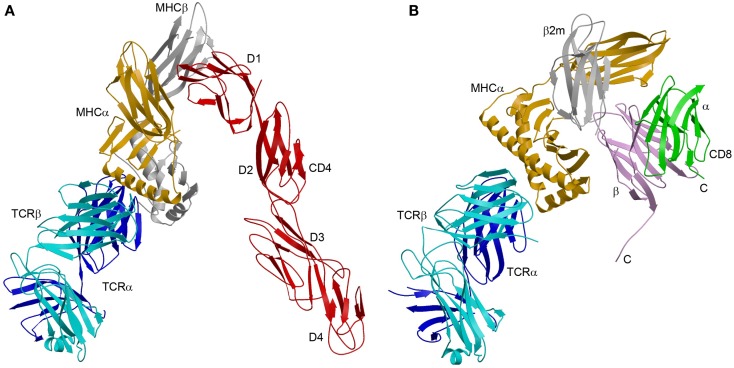
**Comparison of TCR–pMHC–CD4 and TCR–pMHC–CD8 ternary complexes**. **(A)** Crystal structure of a TCR–pMHC–CD4 complex (MS2-3C8–MBP–DR4–CD4) oriented as if the TCR and CD4 molecules are attached to the T cell at the bottom and the MHC class II molecules is attached to an opposing APC at the top (3T0E) (49). TCR α chain, blue; TCR β chain, cyan; MHC α chain, yellow; MHC β chain, gray; CD4, red. **(B)** Hypothetical model of the TCR–pMHC–CD8 complex. The model was constructed by superposing the CD8αβ–H-2D^d^ complex (3DMM) (23) onto a TCR–H-2D^b^ complex (3PQY) through the MHC class I molecule. A portion of the CD8β stalk region was visible in the crystal structure and points toward the T cell membrane. The C-termini of the CD8α and CD8β chains are labeled. The orientation of the TCR–pMHC complex is the same as in **(A)**. TCR α chain, blue; TCR β chain, cyan; MHC α chain, yellow; β_2_m, gray; CD8α, green; CD8β, violet.

The enhanced affinity of this CD4 mutant made possible the crystallization and structure determination of a complete TCR–pMHC–CD4 ternary complex involving a human autoimmune TCR (MS2-3C8), a self-peptide from myelin basic protein (MBP) bound to HLA-DR4, and CD4 (49) (Figure 3A). The TCR–pMHC–CD4 complex resembles a pointed arch in which both TCR and CD4 are tilted rather than oriented vertically. The TCR and CD4 molecules each make an angle of ∼65° with the T cell surface. The apex of the arch is formed by the α2 and β2 domains of HLA-DR4 and the D1 domain of CD4. MS2-3C8 engages MBP via the canonical docking mode of αβ TCRs (51), in which the TCR adopts a central diagonal orientation over pMHC (52, 53). There are no direct contacts between TCR and CD4 (Figure 3A), in agreement with an earlier prediction (47).

The CD4 molecule bound to TCR–pMHC retains the overall extended conformation observed in different crystal forms of unbound CD4 (54), with some hinge-like variability at the D2–D3 junction. The limited segmental flexibility of CD4 implies that any significant variations in overall complex architecture must arise from differences in TCR docking on pMHC.

**Figure 4:**
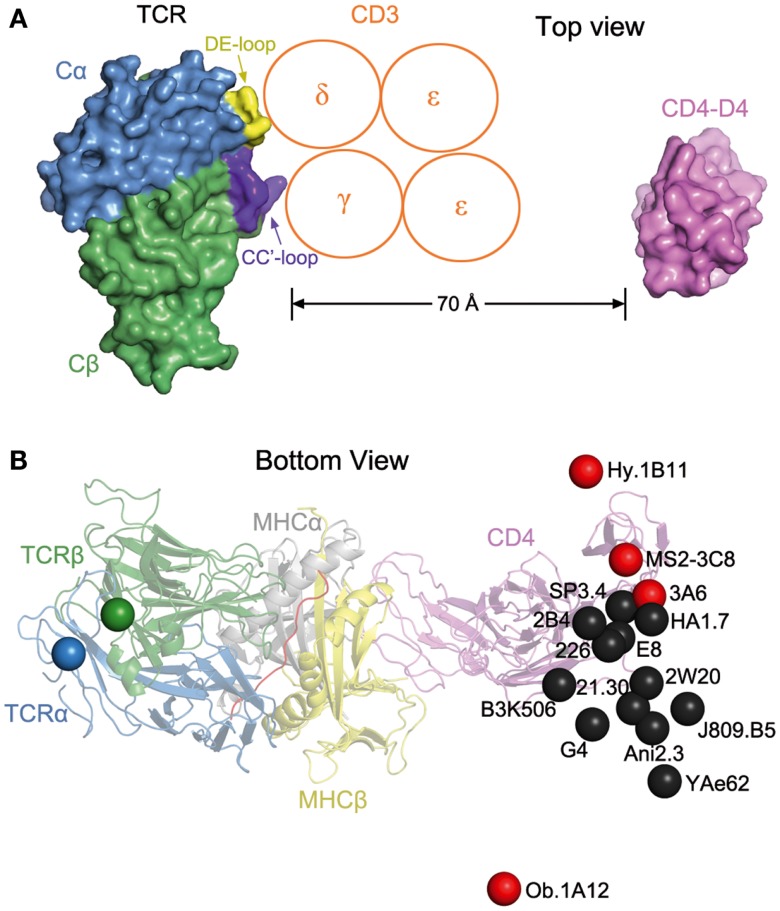
**Orientation of TCR and CD4 in TCR–pMHC–CD4 complexes**. **(A)** Top view of the MS2-3C8–MBP–DR4–CD4 complex (Figure 3A), as if looking down on the T cell. The membrane-proximal TCR Cα/Cβ domains and the CD4 D4 domain are depicted in surface representation. Other domains and pMHC are omitted for clarity. TCR Cα, blue; TCR Cβ, green; CD4 D4, pink. The proposed arrangement of the ectodomains of CD3εγ and CD3εδ (58) is shown in relation to docking sites identified by mutational analyses (56, 58): Cα DE loop (yellow) and Cβ CC′ loop (dark blue). The Ig-like ectodomains of CD3εγ and CD3εδ are drawn as orange circles. In this arrangement, only CD3γ and CD3δ contact the TCR. CD3ε projects away from the TCR, toward CD4. **(B)** Bottom view of the MS2-3C8–MBP–DR4–CD4 complex, as if looking up from inside the T cell. On the left side, the C-termini of the extracellular portions of the α and β chains of TCR MS2-3C8, as defined in the crystal structure (49), are indicated by blue and green spheres, respectively. On the right side, the C-terminus of the extracellular portion of CD4 in the complex with MS2-3C8 and HLA-DR4 is marked by a red sphere labeled MS2-3C8. The right side also shows the predicted position of the C-terminus of CD4 in 15 hypothetical ternary complexes constructed using other TCR–pMHC class II structures [human: HA1.7 (1JH8), Ob.1A12 (1YMM), 3A6 (1ZGL), E8 (2IAM), Hy.1B11 (3PL6), G4 (4E41); SP3.4 (4GG6); Ani2.3 (4H1L); mouse: B3K506 (3C5Z), 2W20 (3C6L), YAe62 (3C60), 21.30 (3MBE); J806.B5 (3RDT); 2B4 (3QIB); 226 (3QIU)]. In each case, the C-terminus of CD4 is marked by a colored sphere labeled with the name of the corresponding TCR. Autoimmune TCRs (MS2-3C8, Ob.1A12, 3A6, Hy.1B11) are red; anti-foreign (HA1.7, B3K506, 2W20, 21.30, YAe62, SP3.4, Ani2.3, J805.B5, 2B4, 226) and anti-tumor TCRs (E8, G4) are black. The TCR–pMHC–CD4 complexes were modeled by superposing each TCR–pMHC class II structure onto the MS2-3C8–MBP–DR4–CD4 complex through the Cα/Cβ domains of the TCRs. The anti-foreign and anti-tumor TCRs (black spheres) form a cluster that mostly excludes the autoimmune TCRs (red spheres), with Hy.1B11, MS2-3C8, and 3A6 on one side of the cluster and Ob.1A12 on the other.

The absence of direct contacts between TCR and CD4 explains how these molecules can simultaneously, yet independently, bind to pMHC. Importantly, the wide separation (∼70 Å) between the membrane-proximal TCR Cα/Cβ module and CD4 D4 domain provides ample room for the placement of TCR-associated CD3εγ, εδ, and ζζ subunits (Figure 4A), which transmit activation signals to the T cell (55). Although no crystal structure is available for the TCR–CD3 complex, mutational studies have identified docking sites for the ectodomains of CD3εδ and CD3εγ, which interact with the TCR through adjacent Cα DE and Cβ CC′ loops, respectively (56–58). Based on this information, CD3εγ and CD3εδ would be situated inside the TCR–pMHC–CD4 arch, wedged between the TCR and T cell membrane (Figure 4A). In the organization of the TCR–CD3 complex proposed by Fernandes et al. (58), only CD3γ and CD3δ contact the TCR, whereas CD3ε projects away from the receptor. The relative proximity of CD3ε to CD4 in the TCR–pMHC–CD4 complex, compared to CD3γ or CD3δ, may confer preference to CD3ε in the ITAM phosphorylation cascade upon Lck recruitment by CD4 (Figure 4A).

The ectodomain of CD3ζ, which is only nine amino acids in length, has not been implicated in interactions with the TCR ectodomain, and so is not shown in Figure 4A. However, mutational analysis of the transmembrane regions of TCR and CD3 subunits has established that CD3ζζ is associated with TCRα in the T cell membrane (59).

It has been proposed that, in resting T cells, CD3ε ITAMs are sequestered in the membrane, and that activation results in ITAM exposure to Src kinases (60). If so, the TCR–pMHC–CD4 structure suggests a possible mechanism by which this may occur. If the TCR shifts from an upright to a tilted orientation upon formation of the TCR–pMHC–CD4 complex (Figure 3A), this movement could potentially drive the CD3 ectodomains, situated inside the TCR–pMHC–CD4 arch, into the T cell membrane. This in turn could cause displacement of CD3ε ITAMs from the membrane and their phosphorylation by Lck.

## The TCR–pMHC–CD4 Complex and Models for TCR Triggering

There is considerable controversy over the mechanism of TCR triggering, and a variety of models have been proposed to explain how pMHC binding to TCR initiates signaling across the T cell membrane (55). Some of these models invoke dimerization (or oligomerization) of CD4 (54), MHC (61), or TCR (57) as a means of enhancing phosphorylation of CD3 ITAMs by increasing the proximity of associated tyrosine kinases. The plausibility of these models can be assessed in terms of the geometrical constraints imposed by the TCR–pMHC–CD4 structure.

The structure of human CD4 D1–D4 in unbound form showed that CD4 molecules form dimers through the D4 domain, at least in the crystal (54). This observation suggested that D4–D4-associated CD4 dimers might contribute to T cell activation by cross-linking TCR–pMHC complexes (54, 62). However, the TCR–pMHC–CD4 structure is incompatible with this idea. In a hypothetical model constructed by superposing the TCR–pMHC–CD4 structure onto the D4–D4-associated CD4 dimer, the distance between the C-termini of the D4 domains and the T cell surface is too far (∼40 Å) to be spanned by the eight-residue stalk region of CD4 (49). Similarly, the finding that some HLA-DR molecules crystallize as dimers (61, 63) suggested a mechanism for T cell triggering in which an MHC class II dimer cross-links two TCRs. However, the CD4-binding site on HLA-DR4 almost completely overlaps the putative HLA-DR dimerization site, which would preclude formation of such MHC class II dimers (49).

Recently, it was proposed that TCRs can dimerize in the T cell membrane via Cα–Cα interactions, and that the resulting juxtaposition of two TCR–pMHC complexes facilitates signaling through the membrane (57, 64). Consistent with this model, the putative site of Cα–Cα dimerization is on the outside of the TCR–pMHC–CD4 arch, opposite the sites mediating TCR–CD3 interactions (Figure 4A) (49). As such, CD4 would not interfere sterically with TCR dimerization through the Cα domain. However, a survey of 22 TCR–pMHC crystal structures failed to reveal any Cα–Cα contacts consistent with biologically relevant TCR dimerization (65). More tellingly, the Cα domain contains two conserved *N*-linked glycans at positions that would preclude the hypothesized TCR dimerization via Cα–Cα interactions.

Several recent studies have demonstrated that physical force applied to the TCR–CD3 complex can activate T cells (66–68). This finding has led to the concept of the TCR as an anisotropic mechanosensor that converts mechanical energy into a biochemical signal upon specific pMHC ligation as a T cell moves over APCs during immune surveillance (66). While it is unknown how pulling on the TCR–CD3 complex can be transduced to the T cell interior, one possibility is that pMHC binding leads to a conformational change in the CD3 cytoplasmic tails, allowing ITAM phosphorylation by Src kinases. This process may be facilitated by the CD4 (or CD8) co-receptor, whose binding to the TCR–pMHC complex could promote dissociation of CD3 ITAMs from the cytoplasmic side of the T cell membrane and their exposure to Lck, as discussed above.

## Co-Receptors and TCR–pMHC Docking Orientation

The TCR–pMHC–CD4 complex provides a basis for understanding how the CD4 and CD8 co-receptors focus TCR on MHC to guide TCR docking on pMHC during thymic T cell selection. Structural studies of numerous (>25) TCR–pMHC complexes have demonstrated remarkable similarities in the overall topology of TCR binding to pMHC, regardless of MHC class I or class II restriction (52, 53). Typically, the TCR is positioned diagonally over the center of the composite surface created by the peptide and the MHC α-helices that flank the peptide-binding groove, with Vα situated over the N-terminal half of the peptide, and Vβ over the C-terminal half, although the exact angle and pitch of TCR engagement vary.

Two competing (though not mutually exclusive) hypotheses have been proposed to explain this roughly conserved diagonal binding mode. The first maintains that co-evolution of TCR and MHC genes has led to specific interaction motifs between the germline-encoded CDR1 and CDR2 loops of TCRs and the α-helices of MHC proteins (53, 69–73). According to the second hypothesis, TCR docking topology is guided by the CD4 and CD8 co-receptors during T cell development in order to achieve intracellular juxtaposition of co-receptor-bound Lck with CD3 ITAMs (11–13, 49, 71, 72, 74, 75). According this view, it is the need for co-receptor function during thymic T cell selection that restricts the geometry of TCR–pMHC recognition and eliminates from positive selection CD4^+^CD8^+^ double-positive thymocytes expressing TCRs unable to engage pMHC in a manner that generates a signal to induce maturation.

The arch-shaped TCR–pMHC–CD4 complex establishes anchor points for TCR and CD4 on the T cell membrane, thereby imposing constraints on the orientation of CD3 relative to Lck associated with CD4 on the cytoplasmic side of the membrane. Figure 4B shows the position of the C-terminus of CD4 observed in the complex with TCR MS2-3C8 and HLA-DR4, as well as the predicted position of the C-terminus of CD4 in hypothetical ternary complexes constructed using 15 other TCR–pMHC class II structures, both human and mouse. Except for the human autoimmune TCR Ob.1A12 (76), the C-termini of CD4 in these modeled complexes are grouped in a loose cluster that includes the C-terminus of CD4 in the MS2-3C8–MBP–DR4–CD4 complex. Differences in the position of the CD4 membrane anchor point are attributable to variations in the diagonal docking topology of the TCR–pMHC complexes, which places CD3εγ and CD3εδ inside the TCR–pMHC–CD4 arch, opposite CD4 (Figure 4A). If the TCR–pMHC docking polarity were reversed (i.e., Vα over the C-terminus of the peptide and Vβ over the N-terminus), CD3εγ and CD3εδ would be positioned outside, rather than inside, the TCR–pMHC–CD4 arch. The much greater distance between CD4-bound Lck and CD3 ITAMs would likely hinder ITAM phosphorylation by Lck, thereby preventing positive selection of T cells bearing TCRs with the reversed polarity, or their activation in the periphery. We therefore propose that the diagonal docking topology of TCR–pMHC complexes reflects not only genetically encoded interactions with MHC (53, 69), but also the requirement to form a ternary complex with the CD4 or CD8 co-receptor that is geometrically competent to deliver a maturation signal to CD4^+^CD8^+^ thymocytes during T cell selection.

Nonetheless, some flexibility must exist within the overall signaling complex to accommodate variations in TCR–pMHC docking geometry that affect the location of anchor points for TCR and CD4 on the T cell membrane (Figure 4B). Given the rigidity of the CD4 ectodomain (49), this flexibility most likely resides in interactions involving the flexible cytoplasmic tails of CD3 and CD4 with Lck, which itself can adopt multiple conformations (77). The flexibility of the cytoplasmic domains of CD3 is supported by circular dichroism analysis and disorder prediction algorithms (78). By both methods, the cytoplasmic domains of CD3ζ, CD3ε, CD3γ, and CD3δ were found to be intrinsically unstructured, random-coil proteins in both monomeric and oligomeric states, and in the presence or absence of lipids.

Of the 16 TCRs in Figure 4B, 4 are autoimmune (Hy.1B11, MS2-3C8, 3A6, Ob.1A12). These TCRs, which were isolated from different multiple sclerosis patients, recognize MBP self-peptides bound to HLA-DQ1 (Hy.1B11), HLA-DR4 (MS2-3C8), HLA-DR2a (3A6), or HLA-DR2b (Ob.1A12). T cells expressing these four autoimmune TCRs escaped negative selection in the thymus yet still retained the ability to productively engage self-antigens in the periphery. The remaining 12 TCRs recognize foreign antigens, such as influenza virus hemagglutinin (HA1.7) and moth cytochrome c (2B4, 226), or tumor antigens (G4, E8). In Figure 4B, the C-termini of CD4 in the 16 TCR–pMHC–CD4 complexes sweep out an arc of ∼70°, with autoimmune TCRs Ob.1A12 and Hy.1B11 at the two extremities. Strikingly, the 12 anti-foreign or tumor-specific TCRs form a relatively tight cluster that effectively excludes the four autoimmune TCRs, with Ob.1A12 on one side of the cluster and Hy.1B11, MS2-3C8, and 3A6 on the other. Because CD4-bound Lck would be positioned differently with respect to CD3 ITAMs inside the T cell, phosphorylation of CD3 ITAMs by Lck may be differentially affected in TCR–pMHC–CD4 complexes involving anti-foreign versus autoimmune TCRs. In this way, the geometry of the TCR–pMHC–CD4 complex could modulate TCR signaling, and thereby directly impact T cell development and autoimmunity.

## Implications for the TCR–pMHC–CD8 Ternary Complex

Although no structure of a TCR–pMHC–CD8 ternary complex has been reported, a hypothetical model may be constructed by superposing the CD8αβ–H-2D^d^ complex (23) onto a representative TCR–pMHC class I complex through the shared MHC class I molecule (Figure 3B). The model is consistent with the idea that the shorter CD8β stalk helps orient the cytoplasmic domains of CD8αβ for their role in signal transduction (23, 79). However, in the absence of structural information on the CD8 stalk, the model cannot establish anchor points for TCR and CD8 on the T cell membrane, as did the TCR–pMHC–CD4 structure (Figure 3A). While the TCR–pMHC–CD8 complex is probably not as conformationally constrained as the TCR–pMHC–CD4 complex, *O*-glycosylation of the CD8 stalk likely restricts its flexibility (36, 42), as discussed above. By limiting the mobility of the MHC-binding head group of CD8 in this way, *O*-glycosylation would impose constraints on the orientation of CD3 subunits relative to CD8-bound Lck, as does the rigid CD4 structure (Figure 4A).

Even with *O*-glycosylation, the proline-rich CD8 stalk is likely to be less stiff than the four tandem Ig-like domains of CD4. This flexibility may help compensate for variations in TCR–pMHC docking geometry to facilitate intracellular juxtaposition of CD8-bound Lck with CD3 ITAMs. The cytoplasmic tails of the CD3 subunits would provide additional flexibility within the overall TCR–pMHC–CD8 signaling complex, just as in the TCR–pMHC–CD4 complex.

## Future Directions

Much more is known about the biophysics of CD8 interactions with TCR–pMHC at the T cell–APC interface than in the case of CD4. For example, whereas *in situ* studies have demonstrated that TCR and CD8 bind cooperatively to pMHC, and that this synergy amplifies peptide discrimination (9), there is no comparable information for CD4. Conversely, whereas the structure of a TCR–pMHC–CD4 complex has been reported (49), that of a TCR–pMHC–CD8 complex remains to be determined.

Until now, most studies of autoimmunity have emphasized reduced TCR affinity for self-pMHC as the main reason autoreactive T cells sometimes escape negative selection in the thymus (80). However, it is also possible that the altered docking topologies observed in autoimmune TCR–pMHC complexes (81) may modulate T cell signaling by altering interactions with CD4 or CD8. In fact, autoimmune TCRs appear to segregate from anti-foreign TCRs in terms of the geometry of the corresponding TCR–pMHC–CD4 ternary complexes (Figure 4B). This hypothesis clearly merits further investigation, particularly in view of an emerging appreciation for the role of TCR docking geometry on T cell signaling (49, 71).

The TCR–pMHC–CD4 structure, in conjunction with mutational data on TCR–CD3 ectodomain interactions, suggests that CD3εγ and CD3εδ are located under the TCR–pMHC–CD4 arch, facing CD4 (Figure 4A). However, the current reality is that we only have nebulous ideas about the molecular architecture of the TCR–CD3 complex. Direct structural analysis of the TCR–CD3 complex will be required to establish anchor points for CD3 and CD4 on the T cell membrane. This information, combined with the known pairings of the transmembrane helices of the TCR and CD3 chains from mutational analysis (TCRα–CD3εδ, TCRα–CD3ζζ, and TCRβ–CD3εγ) (59), will reveal the intracellular organization of CD3 ITAMs relative to each other, and relative to Lck bound to the CD4 or CD8 co-receptor.

## Conflict of Interest Statement

The authors declare that the research was conducted in the absence of any commercial or financial relationships that could be construed as a potential conflict of interest.
